# Prediction model for missed abortion of patients treated with IVF-ET based on XGBoost: a retrospective study

**DOI:** 10.7717/peerj.14762

**Published:** 2023-01-30

**Authors:** Guanghui Yuan, Bohan Lv, Xin Du, Huimin Zhang, Mingzi Zhao, Yingxue Liu, Cuifang Hao

**Affiliations:** 1 Department of Qingdao Medical College, Qingdao University, Qingdao, Shandong, China; 2 Department of Intensive Care Unit, The Affiliated Hospital of Qingdao University, Qingdao, Shandong, China; 3 Department of Reproductive Medicine, The Affiliated Women and Children’s Hospital of Qingdao University, Qingdao, Shandong, China

**Keywords:** IVF-ET, Missed abortion, Machine Learning, Prediction model, XGBoost

## Abstract

**Aim:**

In this study, we established a model based on XGBoost to predict the risk of missed abortion in patients treated with *in vitro* fertilization-embryo transfer (IVF-ET), evaluated its prediction ability, and compared the model with the traditional logical regression model.

**Methods:**

We retrospectively collected the clinical data of 1,017 infertile women treated with IVF-ET. The independent risk factors were screened by performing a univariate analysis and binary logistic regression analysis, and then, all cases were randomly divided into the training set and the test set in a 7:3 ratio for constructing and validating the model. We then constructed the prediction models by the traditional logical regression method and the XGBoost method and tested the prediction performance of the two models by resampling.

**Results:**

The results of the binary logistic regression analysis showed that several factors, including the age of men and women, abnormal ovarian structure, prolactin (PRL), anti-Müllerian hormone (AMH), activated partial thromboplastin time (APTT), anticardiolipin antibody (ACA), and thyroid peroxidase antibody (TPO-Ab), independently influenced missed abortion significantly (*P* < 0.05). The area under the receiver operating characteristic curve (AUC) score and the F1 score with the training set of the XGBoost model (0.877 ± 0.014 and 0.730 ± 0.019, respectively) were significantly higher than those of the logistic model (0.713 ± 0.013 and 0.568 ± 0.026, respectively). In the test set, the AUC and F1 scores of the XGBoost model (0.759 ± 0.023 and 0.566 ± 0.042, respectively) were also higher than those of the logistic model (0.695 ± 0.030 and 0.550 ± 049, respectively).

**Conclusions:**

We established a prediction model based on the XGBoost algorithm, which can accurately predict the risk of missed abortion in patients with IVF-ET. This model performed better than the traditional logical regression model.

## Background

In missed abortion, the embryo stops developing for various reasons, but the dead embryo remains in the uterine cavity (Segawa et al., 2017; Wu et al., 2017). Missed abortion is diagnosed by ultrasound and has an incidence of 8–20% in clinically confirmed intrauterine pregnancy (Fang et al., 2018). Various factors affect embryonic development, including the age of women, genetic factors, endocrine diseases, immune factors, pregnancy infection, pregnancy history, behavioral factors, *etc*. Due to the complexity of the etiology, the pathogenesis of missed abortion is not known (Fang et al., 2018; Guo et al., 2016; Zhao et al., 2017; Zhao et al., 2019; Zhou et al., 2019).

Missed abortion seriously affects the health of the affected women. If the dead embryos are not found on time and remain in the uterine cavity for long, they can cause abnormal maternal blood coagulation, uterine adhesion, disseminated or diffuse intravascular coagulation (DIC), and even threaten the life of the individual (Fang et al., 2018). For women who require assisted reproductive technology, missed abortion causes greater physical and mental harm. Additionally, they and their family have to bear economic and psychological pressure (Hu et al., 2018). IVF-ET is an effective reproductive technology for the clinical treatment of infertility. However, the incidence of missed abortion among individuals treated with IVF-ET can be as high as 18–30% (Qiao & Li, 2013). For couples wanting children, a diagnosis of missed abortion affects individuals emotionally. To reduce such events, high-risk factors need to be identified, and targeted preventive measures need to be provided. Although several studies have screened the high-risk factors of missed abortion (Feng et al., 2021; Jiang, Yang & Luo, 2022), studies targeting infertile people are limited. The method to predict high-risk groups is not well-defined, and therefore, a method for predicting missed abortion for patients treated with IVF-ET needs to be developed. Such a theoretical basis for targeted personalized diagnosis and treatment might enhance the success rate of pregnancy.

Predictive models are widely used in the medical field, and the common modeling methods include traditional logical regression (Lian et al., 2022; Xu et al., 2022) and machine learning (Krenz et al., 2022; Yan et al., 2021). A traditional prediction model is a well-known modeling and analysis method in the field of ART, but its application is limited. First, the accuracy of the model is low. A study constructed a traditional logistic regression model and machine learning model to predict the potential of embryo implantation, and the AUC was 0.66 and 0.74, respectively (Blank et al., 2019). Barnett-Itzhaki evaluated whether the machine learning method is better than the traditional statistical modeling in predicting the outcome of IVF and found that the accuracy is 0.69∼0.90 and 0.34∼0.74, respectively (Barnett-Itzhaki et al., 2020). Second, the method to collect and store medical data has improved considerably, and the data can be integrated and shared in large-capacity information systems. Unlike machine learning, traditional logical regression cannot deal very well with a large number of multi-class features or variables (Altman, 2017). Additionally, the traditional model relies on predetermined equations and does not have the ability of autonomous learning, and thus, it is impossible to use traditional models for building an automated clinical decision-making system to help doctors make decisions (Krittanawong et al., 2017).

Machine learning is the core of artificial intelligence, and its data processing, induction, and synthesis abilities are better than those of other statistical methods. A lifting algorithm based on the regression tree called extreme gradient boosting (XGBoost) is a machine learning hot spot. Because of its short training time and high precision, it is widely used in the medical field (Chen & Guestrin, 2016). The XGBoost algorithm can make up for the limitations of traditional logical regression. It can simulate nonlinear effects and has high efficiency and accuracy. The XGBoost algorithm can perform parallel operations and run large-scale data quickly; it can automatically optimize split nodes and can effectively deal with irregular data with many outliers and missing values. It can also learn independently, and the model constructed is interpretable and flexible (Chen & Guestrin, 2016; Gao et al., 2018). In this study, we integrated many clinical characteristics of IVF-ET patients to construct an XGBoost prediction model for predicting the risk of missed abortion. We then compared this model with the traditional logical regression prediction model to evaluate the prediction performance of the model.

## Methods

### Study design

This retrospective study was approved by the Ethics Committee of the Affiliated Women and Children’s Hospital of Qingdao University (QFELL-YJ-2022–18). As this was a retrospective study, informed patient consent was waived by the Ethics Committee of the Affiliated Women and Children’s Hospital of Qingdao University.

### Participants

Data were collected from 1,017 infertile women who were treated in the Reproductive Center of Qingdao Women’s and Children’s Hospital from September 2019 to May 2022. The inclusion criteria were as follows: (1) Patients who received complete treatment of IVF-ET and completed follow-up in our center; (2) patients with intrauterine pregnancy diagnosed by ultrasound after treatment; (3) patients diagnosed with a singleton pregnancy. The exclusion criteria were as follows: (1) Couples receiving donated sperm or eggs; (2) patients with ectopic pregnancy; (3) patients with multiple pregnancies; (4) pregnancy failure due to special reasons such as medication or trauma; (5) patients who also had severe heart, liver, lung, kidney, and other organ disorders; (6) incomplete information in the patient database. Based on the pregnancy outcome, individuals who had missed abortions were placed in the observation group (*n*  =  340), and those in the normal pregnancy population were placed in the control group (*n*  =  677).

### Diagnostic criteria

The results of ultrasonic examinations showed that missed abortion was associated with one of the following criteria (American College of Obstetricians and Gynecologists, 2018; Huchon et al., 2016): (1) the embryo had a head, a hip length of ≥ seven mm, and lacked a heartbeat; (2) the gestational sac was ≥ 25 mm in diameter without an embryo; (3) for a gestational sac without a yolk sac, there was no heartbeat after two weeks; (4) for a gestational sac with a yolk sac, there was still no heartbeat after 11 days.

### Variables included

The included predictive variables were determined based on those included in published studies and a group discussion among experts. The following data were obtained from medical records: (1) genetic factors, including chromosomal abnormalities in men and women (Feng et al., 2020); (2) female hormone levels, including the level of follicle-stimulating hormone (FSH), luteinizing hormone (LH), estradiol (E2), progesterone (P), testosterone (T), prolactin (PRL), and AMH (Puget et al., 2018; Xie et al., 2021); (3) thyroid hormone levels for serum-free triiodothyronine (FT3), serum-free thyroxine (FT4), thyroid stimulating hormone (TSH), thyroglobulin antibody (TG-Ab), and TPO-Ab (Zhang, Zhao & Zhou, 2018); (4) immune factors, including anticardiolipin antibody (ACA) and antinuclear antibody (ANA) (Yu et al., 2020); (5) coagulation function tests, including prothrombin time (PT), APTT, thrombin time (TT), plasma fibrinogen (FIB), and D-dimer (Dong & Du, 2013); (6) infection factors: leucorrhea test, Chlamydia trachomatis, *Neisseria gonorrhoeae*, TORCH test, *etc*. (Deng, Long & Gao, 2004; Sun et al., 2022); (7) abnormal ovarian structure: ultrasonography examinations showed abnormal number, size, polycystic changes, or space occupying lesions in the ovary (National Collaborating Centre for Women’s and Children’s Health (UK), 2013); (8) uterine structural lesions, such as uterine malformation, leiomyoma, adenomyosis, scar diverticulum, etc. (National Collaborating Centre for Women’s and Children’s Health (UK), 2013); (9) male sperm abnormalities (Dong et al., 2017); (10) other factors, such as the age of men and women, female BMI, fasting blood glucose, blood type, a history of uterine surgery, type of infertility, years of infertility, type of cycle, the number of sinus follicles, the number of eggs obtained, the number of high-quality embryos, the thickness and type of endometrium on the day of transfer, and the quality of the embryo (Van Loendersloot et al., 2014).

### Data processing and analysis

All data were analyzed using the SPSS 20.0 statistical software (IBM SPSS, Inc., Chicago, IL, USA). Continuous variables that followed a normal distribution were expressed as the mean  ± standard deviation. Continuous variables that did not follow a normal distribution were expressed as the median (25th–75th percentile), and classified variables were expressed as frequency (percentage). Based on the data, the *T*-test was conducted for normally distributed continuous variables, the Mann–Whitney U test was conducted for non-normally distributed continuous variables, and the Chi-squared test was conducted for classified variables. All differences between groups were considered to be statistically significant at *P* < 0.05. The independent risk factors were determined by performing a binary logistic regression analysis.

### Prediction methods and model evaluation

All independent risk factors were processed using Python (version 3.0; https://www.python.org/download/releases/3.0/) to construct the prediction model based on XGBoost and traditional logical regression. The train_test_split function of the sklearn package was used to split the dataset into two; 70% of it formed the training dataset, and 30% formed the test dataset (Lapolla et al., 2011). The data from the training set were used to construct the prediction model. The predictability of the constructed model was evaluated using the resampling method. The reference indicators included the AUC and F1 scores. The feature importance function of the better model was used to rank the importance of the variables.

## Results

### Univariate analysis

In total, 1,017 cases were included following the strict standard, including 48 influencing factors. Among these factors, data on the Rh blood group, Chlamydia infection, gonorrhea, *Trichomonas vaginitis*, and the TORCH test were removed because of the large proportion gap between the classification groups. The results for the remaining factors are shown in Tables 1 and 2. In total, 12 indicators with statistical differences were selected. The average age of women and men was higher in the observation group, and there were also more chromosomal abnormalities in this group than that in the control group. Additionally, there were more abnormal ovarian structures, higher PRL, and lower AMH levels, shorter PT and APTT, a higher proportion of positive anticardiolipin antibody (ACA) and antinuclear antibody (ANA), lower serum-free thyroid level, and higher anti-thyroid peroxidase antibody (TPO-Ab)-positive rate among individuals in the observation group compared to that among individuals in the control group.

**Table 1:** Comparison of continuous variables between the observation and control groups.

Variables	Observation group (*n* = 340)	Control group (*n* = 677)	*t*/*z*	*P*
Female age, years	34.11 ± 4.65	32.12 ± 3.75	−7.378	<0.001^***^
Male age, years	33.58 ± 5.23	32.95 ± 4.37	−2.046	0.041^*^
BMI, kg/m^2^	23.53 ± 2.35	23.17 ± 3.28	−1.775	0.076
Years of infertility	3.60 ± 1.47	3.38 ± 2.28	−1.606	0.109
FSH, mIU/mL	7.34 ± 2.19	7.27 ± 3.34	−0.360	0.719
LH, mIU/mL	5.65 ± 3.67	6.23 ± 5.97	1.649	0.099
PRL, ng/mL	23.15 ± 16.38	20.48 ± 9.71	−3.252	0.006^**^
E2, pmol/l	176.65 ± 77.67	187.10 ± 142.22	1.251	0.211
T, ng/mL	0.75 ± 0.26	0.78 ± 0.40	1.378	0.168
P, nmol/l	0.72 ± 0.39	0.78 ± 0.61	1.701	0.089
AMH, ng/mL	2.78 ± 1.48	3.58 ± 2.78	4.964	<0.001^***^
D-Dimer, mg/L	0.25 ± 0.16	0.25 ± 0.25	−0.128	0.882
PT, s	11.16 ± 0.48	11.26 ± 0.80	2.047	0.041^*^
APTT, s	24.50 ± 2.32	25.32 ± 3.34	4.067	<0.001^***^
TT, s	18.05 ± 0.67	18.00 ± 1.48	−0.507	0.612
FIB, g/L	2.67 ± 0.38	2.71 ± 0.89	0.823	0.411
ESR, mm/h	13.34 ± 5.69	12.65 ± 7.89	−1.443	0.149
FBG, mmol/l	5.00 ± 0.45	4.94 ± 0.48	−1.770	0.077
FT3, pmol/l	4.80 ± 0.45	4.83 ± 0.53	0.837	0.403
FT4, pmol/l	15.60 ± 1.56	15.89 ± 2.36	2.041	0.042^*^
TSH,µIU/mL	2.09 ± 0.72	2.13 ± 1.21	0.603	0.547
Left basal sinus follicle	7.11 ± 4.01	7.56 ± 4.29	1.596	0.111
Right basal sinus follicle	7.58 ± 4.48	7.77 ± 4.21	0.696	0.487
Total number of eggs obtained	10.24 ± 3.79	9.82 ± 4.54	−1.468	0.142
Endometrial thickness in transfer date, mm	10.26 ± 1.73	10.34 ± 1.60	0.752	0.452
Number of embryos implanted	2 [1,2]	1 [1,2]	−1.000	0.317
Number of high-quality embryos	3 [2,5]	3 [2,4]	−1.323	0.186

**Notes.**

t statistical value of *t*-test z degree of deviation from variance

* *P* < 0.05.

** *P* < 0.01.

*** *P* < 0.001.

**Table 2:** Comparison of classified variables between the observation and control groups.

Variables	Classification	*n* (%)		*χ* ^2^	*P*
		Observation group	Control group		
Cycle type	Fresh embryo transfer	190 (55.9%)	396 (58.5%)	0.632	0.427
	Frozen embryo transfer	150 (44.1%)	281 (41.5%)		
Chromosome abnormality	NO	289 (85.0%)	605 (89.4%)	4.056	0.044^*^
	YES	51 (15.0%)	72 (10.6%)		
Infertility type	Secondary infertility	147 (43.2%)	286 (42.2%)	0.091	0.763
	Primary infertility	193 (56.8%)	391 (57.8%)		
Male sperm abnormality	NO	250 (73.5%)	513 (75.8%)	0.609	0.435
	YES	90 (26.5%)	164 (24.2%)		
Abnormal ovarian structure	NO	265 (77.9%)	573 (84.6%)	6.999	0.008^**^
	YES	75 (22.1%)	104 (15.4%)		
Abnormal uterine structure	NO	257 (75.6%)	528 (78.0%)	0.742	0.389
	YES	83 (24.4%)	149 (22.0%)		
History of uterine cavity surgery	NO	274 (80.6%)	559 (82.6%)	0.600	0.439
	YES	66 (19.4%)	118 (17.4%)		
ABO blood group	A	111 (32.6%)	199 (29.4%)	3.670	0.299
	B	93 (27.4%)	223 (32.9%)		
	O	100 (29.4%)	181 (26.7%)		
	AB	36 (10.6%)	74 (10.9%)		
ACA	(-)	321 (94.4%)	661 (97.6%)	7.083	0.008^**^
	(+)	19 (5.6%)	16 (2.4%)		
ANA	(-)	330 (97.1%)	669 (98.8%)	4.030	0.045^*^
	(+)	10 (2.9%)	8 (1.2%)		
TG-Ab	(-)	281 (82.6%)	568 (83.9%)	0.257	0.612
	(+)	59 (17.4%)	109 (16.1%)		
TPO-Ab	(-)	233 (68.5%)	542 (80.1%)	16.592	<0.001^***^
	(+)	107 (31.5%)	135 (19.9%)		
Cleaning degree of the vagina	I°	90 (26.5%)	200 (29.5%)	2.612	0.455
	II°	219 (64.4%)	432 (63.8%)		
	III°	29 (8.5%)	42 (6.2%)		
	IV°	2 (0.6%)	3 (0.4%)		
VVC	NO	337 (99.1%)	672 (99.3%)	0.060	0.807
	YES	3 (0.9%)	5 (0.7%)		
Quality of implanted embryos	A	311 (91.5%)	633 (93.5%)	1.400	0.237
	B	29 (8.5%)	44 (6.5%)		
Endometrial type in implant date	A	17 (5.0%)	69 (10.2%)	9.468	0.050
	A-B	10 (2.9%)	12 (1.8%)		
	B	10 (2.9%)	10 (2.8%)		
	B-C	33 (9.7%)	55 (8.1%)		
	C	270 (79.4%)	522 (77.1%)		

**Notes.**

n number of patients *χ*^2^ Chi-Squared test

* *P* < 0.05.

** *P* < 0.01.

*** *P* < 0.001.

### Binary logistic regression analysis

A binary logistic regression model was established with pregnancy outcomes as the dependent variable and the statistically significant variables mentioned above as independent variables. The results are shown in Table 3. The independent influencing factors of missed abortion included female age, male age, abnormal ovarian structure, PRL, AMH, APTT, ACA, and TPO-Ab.

**Table 3:** The results of the binary logistic regression analysis.

Variables	*B*	*SE*	*OR*	*95% CI*	*P*
Female age	0.188	0.028	1.207	1.143∼1.275	<0.001^***^
Abnormal ovarian structure	0.622	0.185	1.863	1.295∼2.679	0.001^**^
PRL	0.020	0.007	1.020	1.006∼1.035	0.006^**^
AMH	−0.121	0.037	0.886	0.824∼0.952	0.001^**^
APTT	−0.059	0.025	0.943	0.898∼0.990	0.018^*^
ACA	0.903	0.377	2.468	1.180∼5.163	0.016^*^
TPO_Ab	0.594	0.162	1.812	1.318∼2.490	<0.001^***^
Male age	−0.115	0.024	0.891	0.851∼0.934	<0.001^***^

**Notes.**

B beta regression coefficient SE standard error OR odds ratio *CI* confidence interval

* *P* < 0.05.

** *P* < 0.01.

*** *P* < 0.001.

### Construction and evaluation of the prediction model

**Figure 1:**
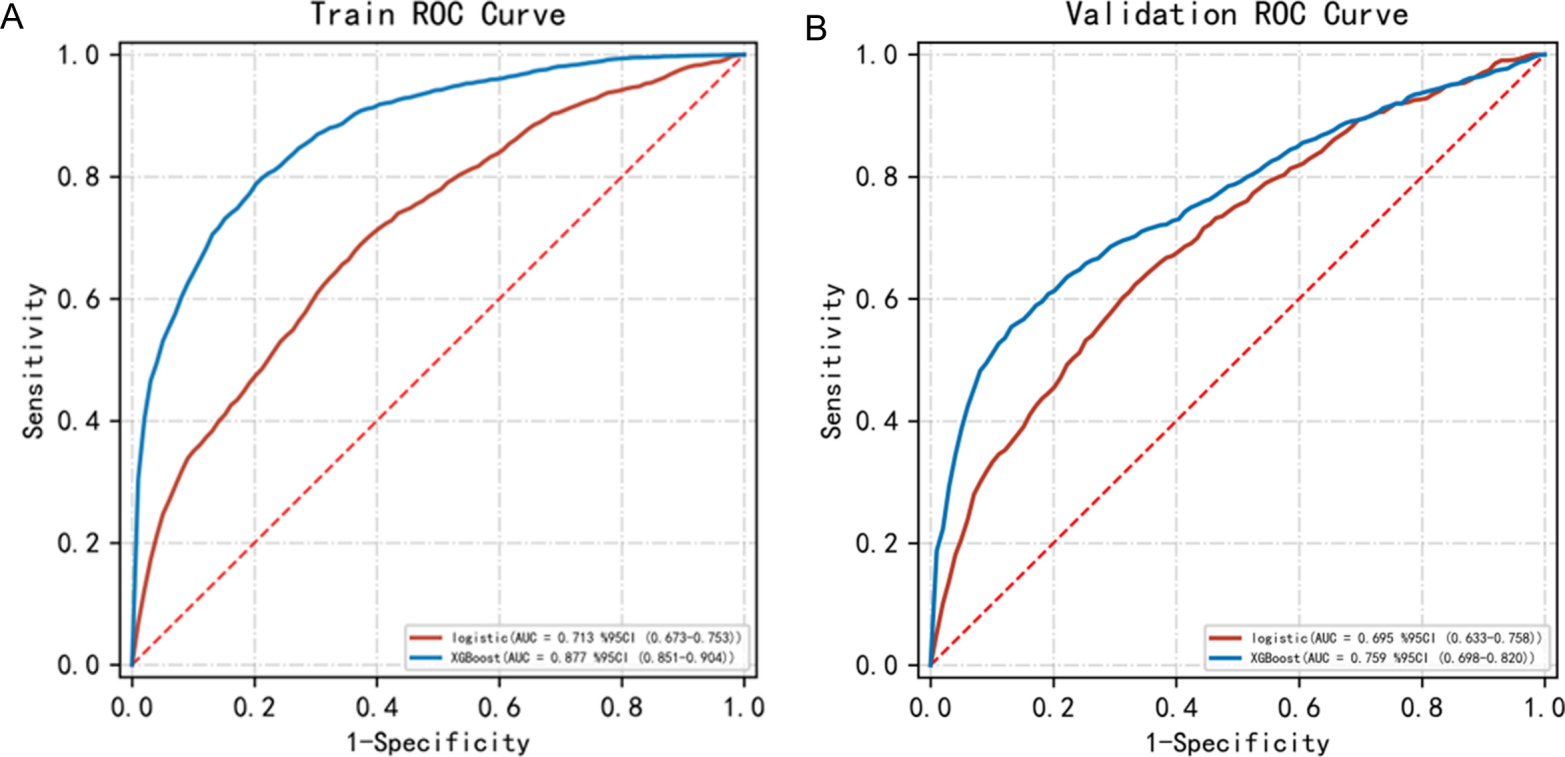
The ROC curves of the two models. (A) The AUC of the logistic model *vs.* the AUC of the XGBoost model (0.713 *vs* 0.877) in the training set. (B) The AUC of the logistic model *vs.* the AUC of the XGBoost model (0.695 *vs* 0.759) in the test set.

**Figure 2:**
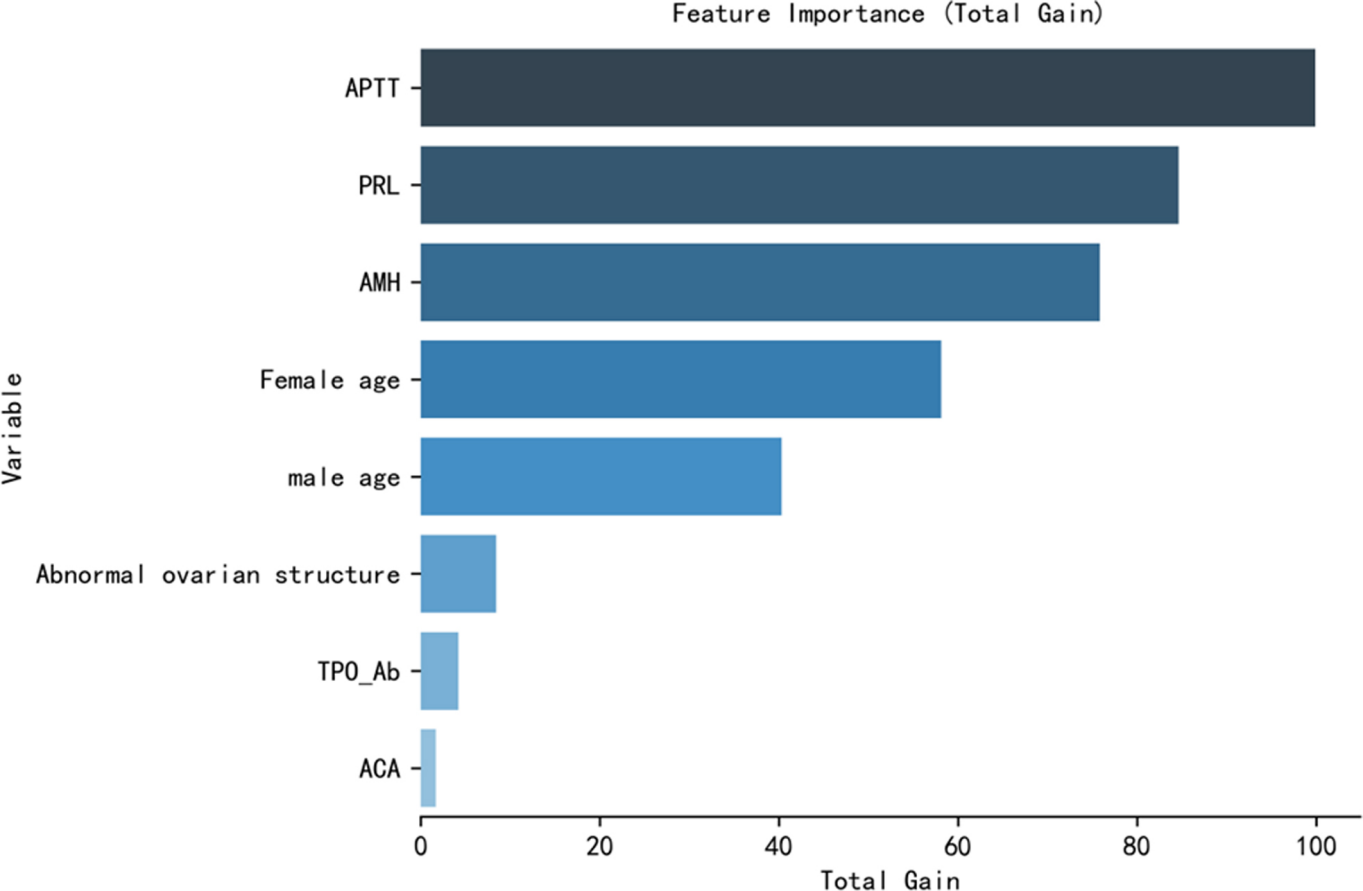
Feature importance of the variables in the XGBoost model. The most important influencing factor was activated partial thromboplastin time (APTT), followed by prolactin (PRL), anti-Müllerian hormone (AMH), female age, male age, abnormal ovarian structure, thyroid peroxidase antibody (TPO-Ab), and anticardiolipin antibody (ACA).

We constructed the prediction model based on the influencing factors presented in Table 3. The hyperparameters used in the XGBoost model included the following: objective-binary: logistic, learning rate = 0.01, max depth = 4, min child weight = 2, and reg lambda = 1. The hyperparameters used in the logical regression model included the following: Regularization factor (C) = 1, max iter = 100, penalty = l2, and tol = 0.0001. The AUC score and the FI score were used to evaluate the performance of the XGBoost model and the logistic regression model (Table 4). The AUC score of the XGBoost model (0.877 ±0.014) was significantly higher than that of the logistic model (0.713 ±0.013). The ROC curves of the two prediction models are shown in Fig. 1A. The F1 score of the XGBoost model (0.730 ±0.019) was also significantly higher than that of the logistic model (0.568 ±0.026). The XGBoost model performed better than the logistic model. The predictability of the two models was evaluated by the resampling method, and the XGBoost model performed better (Table 5 and Fig. 1B). Therefore, the ranking of importance was also based on the XGBoost model (Fig. 2).

**Table 4:** The results of the training set.

	Model	AUC (95% CI)	Accuracy	Sensitivity	Specificity	*F*1 Score
Mean	logistic	0.713 (0.673∼0.753)	0.668	0.657	0.676	0.568
SD	logistic	0.013 (0.014∼0.013)	0.021	0.093	0.073	0.026
Mean	XGBoost	0.877 (0.851∼0.904)	0.802	0.795	0.807	0.730
SD	XGBoost	0.014 (0.015∼0.012)	0.021	0.038	0.045	0.019

**Notes.**

AUC area under the curve *CI* confidence interval

*F*1 Score: harmonic average of precision and recall.

**Table 5:** The results of the test set.

	Model	AUC (95% *CI*)	Accuracy	Sensitivity	Specificity	*F*1 Score
Mean	logistic	0.695 (0.633∼0.758)	0.635	0.659	0.657	0.550
SD	logistic	0.030 (0.032∼0.028)	0.042	0.074	0.065	0.049
Mean	XGBoost	0.759 (0.698∼0.820)	0.705	0.587	0.861	0.566
SD	XGBoost	0.023 (0.026∼0.021)	0.021	0.071	0.045	0.042

**Notes.**

AUC area under the curve CI confidence interval

*F*1 Score: harmonic average of precision and recall.

## Discussion

Assisted reproductive technology has been used in the clinic for many years. IVF-ET technology is also the last hope of many infertile couples. Although the clinical pregnancy rate of patients treated with IVF-ET can increase by up to 46.9%, the live birth rate is very low, around 38.1% only (Sunderam et al., 2017). Missed abortion decreases the success rate of pregnancy and causes deep emotional disturbance among patients (Zhang et al., 2021). Identifying the clinical influencing factors of missed abortion after IVF-ET can facilitate etiological treatment. In this study, we determined eight independent influencing factors.

We found an abnormal coagulation function, such as the shortening of APTT and PT, in women with missed abortions, suggesting the hypercoagulable state of the blood. This might be because the hypercoagulable state of the blood can selectively affect blood circulation in the uterus and placenta, form microthrombus in the placenta, cause a local placental infarction, decrease placental blood supply, and cause ischemia and hypoxia in the embryo and fetus, thus arresting the growth of the embryo (Dong & Du, 2013). Therefore, if blood hypercoagulability is found before or during early pregnancy, appropriate exercise can be recommended to promote blood circulation along with drug treatment, such as heparin.

The hypothalamic-pituitary-ovarian axis regulates the complex endocrine system. Abnormalities in any part of the axis can lead to adverse pregnancy outcomes. In this study, we found that the level of PRL also increased significantly in the observation group, and hyperprolactinemia was common in pituitary dysfunction or space-occupying lesions in the pituitary. An increase in prolactin levels can inhibit the synthesis and release of gonadotropin, adversely affect the development of follicles and embryos, and cause ovulation disorders, resulting in infertility or missed abortion (Grigg et al., 2017). Additionally, the level of AMH in such cases decreases in the placenta and is often accompanied by structural lesions in the ovary. AMH is negatively correlated with the risk of early spontaneous abortion (Tarasconi et al., 2017), and the structural changes in the ovary can affect its function. Progesterone is required for the successful implantation of fertilized eggs and pregnancy, and proper functioning of the ovary is essential for normal pregnancy (Patel et al., 2017). Hence, when the PRL level increases due to pituitary adenomas, or the ovarian function decreases due to substantial ovarian lesions, patients need surgical resection of the lesions and postoperative adjuvant therapy with drugs.

The age of the individual strongly affects the success of pregnancy. As the age of a woman increases, the quality of oocytes decreases, leading to errors during oocyte meiosis, the formation of aneuploidy, chromosome translocation, inversion, *etc*. Embryos formed by such gametes are at a greater risk of spontaneously stopping development (Hansen, 1986; Qiao & Yang, 2017). The results of our study not only confirmed the above-mentioned findings but also showed that male age is an important influencing factor. Some studies have shown that the male sperm quality decreases with an increase in male age, and the probability of gene mutation increases (Brahem et al., 2011; Cooper et al., 2010). In our study, sperm abnormality did not affect the results. This might be because the standard of abnormal sperm was defined based on a clinical diagnosis, such as oligospermia, asthenospermia, and sperm deformity, rather than on the quality of sperm used for *in vitro* fertilization. Although China has implemented the three-child policy, the reproductive desire of young women has decreased, and the proportion of conception among relatively older women in China has increased. Therefore, improving the early pregnancy monitoring of elderly patients receiving ART is important.

We also found that the level of FT4 in women with embryo termination was lower than that in normal people, and the positive rate of TPO-Ab was significantly higher, suggesting that thyroid dysfunction might affect embryos adversely. Hypothyroidism might cause adverse effects, such as embryo termination and fetal malformations (Alexander et al., 2017). TPO-Ab and TG-Ab are specific indicators of thyroid autoimmunity. Abnormal levels of TPO-Ab and TG-Ab can cause autoimmune hypothyroidism. Some studies (Grossmann et al., 2013; Ji, Wang & Song, 2019) have shown that positive TPO-Ab in women during early pregnancy increases the risk of abortion. Therefore, pre-pregnancy thyroid function should be examined, and appropriate treatment should be administered to women diagnosed with thyroid diseases and abnormal laboratory indices; if necessary, multi-disciplinary treatment (MDT) should be provided.

The last influencing factor we discuss in this study is the immune factor. ACA and ANA are human autoimmune antibodies. We found that the level of these two antibodies was higher in women with missed abortions. ACA might act on the membrane phospholipids of placental vascular endothelial cells and platelets in the early stage of pregnancy, block prostacyclin synthesis, and lead to placental embolism. ANA might influence DNA replication and immune abnormalities (Yu et al., 2020). Only a positive laboratory test for ACA or ANA may not cause missed abortion. Usually, when patients have symptoms, they should be administered timely treatment. Some researchers have found that for ACA-positive patients, prednisone and low-dose aspirin therapy can improve pregnancy outcomes (Zhu et al., 2013).

Based on the above factors, we constructed an XGBoost-based missed abortion risk prediction model for patients treated with IVF-ET. The results showed that the prediction performance of the model was better than the prediction performance of the traditional logical regression model. Although many researchers have studied the risk factors for missed abortion, Yi et al. (2016) analyzed the relationship between ultrasound factors and EPL based on the data collected from initial transvaginal ultrasonography scans of 2,601 females with viable singleton pregnancies who underwent IVF-ET. Using these data, they designed a logistic model to predict EPL. Kapfhamer et al. (2018) predicted the loss of pregnancy by measuring the average sac diameter and head-hip length after IVF. Puget et al. (2018) predicted early pregnancy outcomes based on serial human chorionic gonadotropin (hCG) and progesterone levels. The input variables of the prediction models mentioned above were few and focused on one aspect. However, our study integrated 48 possible high-risk factors and analyzed eight independent risk factors through binary logistic regression. We established a comprehensive prediction model and considered the influence of multiple factors to improve the prediction performance of the model. Additionally, the influencing factors included by some researchers were not commonly detected. Yang et al. (2021) detected serum fibroblast growth factor 21 (FGF21) and fatty acid binding protein 4 (FABP4) levels of missed abortion by performing an enzyme-linked immunosorbent assay. Gao et al. (2021) collected fecal specimens from patients and extracted bacterial DNA, and performed bacterial chip assays. They found that a high abundance of Actinobacteria was one of the high-risk factors for missed abortion (Gao et al., 2021). The variables included in our study are routine testing items in the process of IVF-ET treatment. They are easy to extract from the clinical database, either for the next step of external verification or multicenter applications. Most applications of XGBoost prediction models are focused on the automation technology field and are uncommon in the medical field. Qiu et al. (2019) selected age, AMH, BMI, duration of infertility, previous live birth, previous miscarriage, previous abortion, and the type of infertility as predictors to develop four machine learning models to predict live birth. They found that XGBoost provided the most accurate prediction (AUC of the training dataset = 0.74 ± 0.02, AUC of the test dataset = 0.73) on the cumulative live birth chance for IVF cycles (Qiu et al., 2019). We also used XGBoost to construct a prediction model for missed abortions. Besides age and AMH, the coagulation level, thyroid dysfunction, and immune factors caused an adverse pregnancy outcome. Our XGBoost model had high accuracy (AUC of the training dataset = 0.877 ±0.014, AUC of the test dataset = 0.713 ±0.013) and might be used as a theoretical basis for the prevention of missed abortion.

Along with our prediction model, when predicting the risk of missed abortion, clinicians should identify high-risk factors in advance, perform personalized prevention and treatment of these high-risk factors before IVF-ET treatment and during early pregnancy, and monitor patients after confirming clinical pregnancy to avoid a missed diagnosis, which might cause embryos to remain in the uterus for a long time.

With the development of big data, similar prediction models based on machine learning might be constructed by more researchers. These studies might effectively deal with massive clinical databases and use an unbiased method to identify new information variables that are not easy for clinicians to find, to increase the convenience for personalized diagnosis and treatment platforms. However, its application still has some limitations. First, machine learning lacks human emotion and cannot completely replace the human brain in making decisions. Clinicians need to use it as a theoretical basis to make decisions according to the actual situation of the patients. Second, there is a problem of information leakage. Constructing a model requires patient information. Our health system needs to be improved and fortified to ensure the privacy and security of patients.

## Limitations

One limitation of this study was that the inclusion index did not reach an ideal state. Embryonic chromosomes, bad living habits, a history of close contact with pets, and environmental factors were not included because this was a retrospective study. Some of the clinical data used had certain limitations, and standardizing the subjective indicators was challenging. A more accurate forecasting model needs to be constructed with all the indicators. Another limitation was that the study population included individuals from a small area and lacked geographical and ethnic variation. Future studies should consider including data from multiple medical centers for the external verification of the model.

## Conclusions

To summarize, we found that the independent influencing factors of missed abortion included female age, male age, abnormal ovarian structures, PRL, AMH, APTT, ACA, and TPO-Ab. We constructed a prediction model based on the XGBoost algorithm, which could be used to accurately predict the risk of missed abortion in patients with IVF-ET. This model performed better than the traditional logical regression model. The findings of this study might provide a theoretical basis for preventing abortion among women who are at a high risk of missed abortion.

##  Supplemental Information

10.7717/peerj.14762/supp-1 Data S1 Raw Data

## Additional Information and Declarations

### Data Availability

The following information was supplied regarding data availability: The raw data are available in the Supplemental File.
